# Food security of people with celiac disease in the Republic of Moldova through prism of public policies

**DOI:** 10.3389/fpubh.2022.961827

**Published:** 2022-10-03

**Authors:** Rodica Siminiuc, Dinu Ṭurcanu

**Affiliations:** ^1^Department of Food and Nutrition, Faculty of Food Technologies, Technical University of Moldova, Chişinău, Moldova; ^2^Doctoral School of Technical University of Moldova, Chişinău, Moldova

**Keywords:** public policy, celiac disease, gluten free products, level of care, food security, Republic of Moldova

## Abstract

Food security is an important lever for the implementation of rights-based legislation, policies, and programs, while being a public health and socio-economic priority. Foodborne illnesses have a major impact on public health, and nutritional interventions are essential therapeutic strategies to combat them. Gluten ingestion has been linked to several clinical disorders, collectively called gluten-related disorders. The most serious of these is celiac disease. The only way to treat celiac disease is to stick to a gluten-free diet for life. Following a strict diet is also the only way to prevent the long-term consequences of the disease. Public policies are essential to ensure the food security of people with gluten-related disorders. The aim of the research is to assess the level of care for people with celiac disease in the Republic of Moldova, in terms of public policies, to ensure a sustainable sector that effectively satisfies the food security of people with disorders associated with gluten consumption. To assess the level of care for people with gluten-related disorders, the working algorithm was taken, with reference to global public policies in support of people with celiac disease, developed and validated by Falcomer et al., Focused on 6 items. The results of the study showed that the Republic of Moldova does not have adequate policy support to ensure food security for people with gluten-related disorders, which poses major challenges and, as a result, may increase the complications of these problems.

## Introduction

The flexible concept of food security, which emerged in the 1970s in a time of global food crisis, has undergone multiple changes and interpretations, moving from a definition focused on food production to one focused on nutrition. Food security is considered to exist when all people always have physical, social and economic access to sufficient, safe and nutritious food that meets their dietary needs and preferences for an active and healthy life (1–4). Food security is a rather complex notion and focuses on four important dimensions, to which the fifth—sustainability—has subsequently been anchored (Supplementary Figure 1).

Food security has been and remains a priority, but also a key component of the human development paradigm, as access and the right to food are essential for building human capacity (5). At the junction of the right to food and health, food security creates cross-cutting opportunities for the implementation of rights-based legislation, policies, and programs. The interconnection between food and nutrition security requires a simultaneous approach to address the challenges associated with health issues (6). Food security is a socio-economic and public health priority. Foodborne illnesses have a major impact on public health (7). Nutritional interventions are essential therapeutic strategies for combating many chronic diseases, but limited access to varied and nutritious foods can hinder efforts (6, 8).

### Gluten-related disorders

Nutritional therapies aim to address the symptoms and various ailments by examining and changing lifestyle factors and eating habits (9). For some diseases, nutritional therapy is the only possible treatment, which often needs to be followed for the rest of your life. Gluten ingestion has been linked to a number of clinical disorders, collectively called gluten-related disorders, which have gradually emerged as an epidemiologically relevant phenomenon (10) (Supplementary Figure 2).

The most severe of these disorders is celiac disease (CD), an autoimmune disease that affects the small intestine. Gluten ingestion leads to the destruction of enterocyte villi in affected patients. Gluten is made up of protein fractions present in certain cereals (wheat, barley and rye) (11, 12). Multiple studies targeting the immune response to gluten have shown that gliadin, the alcohol-soluble glycoprotein fraction of gluten, is responsible for the adverse reaction to gluten. Gliadins are difficult to digest due to their chemical complexity. From the partial digestion of glutamine and proline fragments are obtained, capable of revoking an inflammatory response with the destruction of intestinal epithelial cells (13, 14).

CD is found in 1–2 people out of 100 worldwide. In the European Union alone, at least 5 million people are affected by CD. Epidemiological data indicate that for every child diagnosed with CD there are at least seven undiagnosed (15). In patients with CD, gluten ingestion triggers chronic small bowel damage. Morphological changes of the intestinal mucosa led to malabsorption syndrome. The only possible way to treat celiac disease is a lifelong gluten-free diet, which improves the clinical picture, normalizes the level of antibodies, and restores the damaged intestinal mucosa. Following a strict diet is also the only way to prevent the serious effects of the disease in the long run. A gluten free diet (GFD) means strict elimination of all gluten-containing products, such as wheat, barley, rye, but also cereal products (e.g., semolina, durum, spelled, triticale, and malt) (13, 16, 17). Studies in the field have shown that the rate of adherence to a GFD varies from 44 to 90% in patients with CD. The food security of the followers of the GFD is affected by multiple barriers, namely: limited availability of products; high cost; insufficient labeling; risk of cross-contamination; lack of knowledge and information about celiac disease and gluten-free diet; psychological factors in celiac patients, etc. (18). All this, in turn, can affect the emotional state and quality of life of people with CD (16, 19).

### Public policies for governing gluten-free products

Public policies influence how people, sectors and institutions interact with each other and provide incentives to improve food security. A favorable environment has several dimensions that policy can influence, including food demand, production and access policies, etc. Public policies are essential because market mechanisms alone cannot provide all the resources needed to produce, store and distribute food along the value chain, nor can they provide the necessary institutions and regulations to support fair and secure food systems (20, 21). Public policies are key to ensuring the food security of people with gluten-related disorders (22). The legislation governing gluten-free labeled food products is represented by international, European, and American standards (Supplementary Figure 3).

The Codex Alimentarius includes all international standards and guides to good practice in the food industry that ensure the safety and health of the consumer in his relationship with food. According to European legislation and Codex Alimentarius, a food can only be labeled “*gluten free”* if it contains <20 ppm gluten (20 mg gluten/kg) in the final product. Also, a food labeled “*with very low gluten content”* may be labeled as such if it contains <100 ppm gluten (100 mg/kg) in the final product. European legislation does not require external audits, focusing on the producer's responsibility and leaving it to him to demonstrate at all times that the product labeled “gluten-free” contains <20 ppm gluten (23, 24).

European Regulation 1169/2011, which regulates good information regarding the ingredients contained in packaged foods, provides the basis for ensuring consumer protection in relation to foods on the market. This regulation requires transparency regarding the ingredients contained as well as the legibility of the information on the product labels (25).

European Regulation 828/2014 specifically regulates the correct labeling and information of consumers regarding the gluten content of packaged foods. Food and Drug Administration (FDA) 101.91 Gluten free labeling food, establishes criteria for “gluten-free” labeling for fermented and hydrolyzed foods, as well as for products that include fermented or hydrolyzed components (26).

The European License System (ELS) is the standard by which gluten-free products are certified (those products bearing the Crossed Grain logo on the packaging). The standard is a sign of consumer safety, integrity and trust in the product and is seen as the most effective way to communicate that a product is safe for the GFD (27). Most national health organizations in Central European countries have diverse systems and approaches for patients with celiac disease, providing information on patients' rights, specific laws, administrative regulations and lists of services (15).

The Republic of Moldova is located in the South-Eastern part of Europe. In the North, East and South it borders Ukraine, and in the West-Romania. It covers an area of 33.8 thousand km^2^. The stable population is about 3.5 million, which is about 0.05% of the world's population. It has a low- and middle-income economy, being considered the poorest country in Europe (28–30). Reports on food security in the Republic of Moldova attest to relatively low overall levels (30). For people with disorders associated with gluten consumption, this criterion was and continues to be quite a sensitive issue, being affected by the Covid-19 pandemic (31), but also by the crisis of the war in Ukraine.

The purpose of the research is to evaluate the food security of people with disorders related to gluten consumption in the Republic of Moldova through the lens of public policies.

## Materials and methods

To assess the level of care for people with gluten-related disorders, the working algorithm was taken, with reference to global public policies in support of people with CD, developed and validated by Falcomer et al. (22).

### The method of Falcomer et al.

The respective methodology was developed based on the critical examination of the literature with reference to the diagnosis and monitoring of CD and GFD; Challenges to dietary adherence and food procurement, particularly for low-income populations; Studies with reference to the well-being of people with CD. The argumentation and deliberation of the items fell to a commission made up of 5 experts in the field from the University of Brasilia and the Federal University of Goia. Accordingly, the following question tool was developed:

1. Does the Republic of Moldova Have Regulations Regarding Packaged Industrial Food Products for People With CD?
2. Does the Country Have Regulations Regarding Unpackaged Meals and Food for People With CD?
3. Is There a Specialized Healthcare Service for Celiac Patients?
4. Is There a Government Food Allowance and / or a Financial Incentive for Patients With CD?
5. Is there a GF certification program for manufactured products for people with GD?
6. Does the Country Have a National CD Association?

### Scoring and quantitative analysis

Responses, collected through Google search, for each WHO member country, were dichotomously categorized and recorded as “Yes” or “No” using a standardized table containing the above-mentioned questions. To form the Public Policy Score with reference to celiac disease (PPSCD), for each “Yes” recorded in response to the selected items, 1 point was assigned, and for each “No” zero points were assigned. Therefore, the result varied on a scale from 0 to 6 points. The score recognizes the instrument's criteria as equally important to complete patient care. According to the working algorithm, depending on the accumulated points, characteristics were assigned: high level of assistance ≥5 points; moderate: 3–4 points; low: 1–2 points; zero: no point.

To evaluate the PPSCD in the Republic of Moldova, the 6-question instrument proposed by Falcomer et al., was taken, using the same evaluation procedure. The answers for each question were searched independently by 2 investigators and later compared.

International and national normative acts / documents were sought regarding the labeling and marketing of gluten-free industrial food, GF catering services (in schools, kindergartens, hospitals, and catering establishments), in support of health services, food allowance and financial incentives for people with celiac disease and the existence of a national celiac disease association.

The search for international regulations was done through the Google search engines and the official pages of the agencies in the field of food security. The search for national normative acts was carried out on the official pages of the institutions of the Republic of Moldova. The uncertainty of some aspects also necessitated discussions with researchers, specialists in the field of diseases associated with gluten consumption from the State University of Medicine and Pharmacology of the Republic of Moldova.

## Results

Points were assigned for each question according to the working methodology adopted. For questions 1, 2, 5 and 6, no regulations or PPs were identified, and the score given was zero, respectively (Table 1). As the national celiac disease protocol (although not updated) only covers children, and other specialized care services for adults diagnosed with CD were not identified, the score for item three was 0.5 points. Patients diagnosed with CD receive financial allowances approved by the Government, according to the general recommendations for people with disabilities, and can benefit, once a year, from rehabilitation (32). Respectively, for item four, 1 point was awarded.

**Table 1:** The public policy score for celiac disease (PPSCD) in the Republic of Moldova.

	**Items used to assess the level of care for people with celiac disease**	**Answer choices and the score awarded**	
		**Yes**	**No**
1.	Does the Republic of Moldova have regulations regarding packaged industrial food products for people with MC?		0
2.	Does the country have regulations regarding unpackaged meals and food for people with MC?		0
3.	Is there a specialized healthcare service for celiac patients?	0.5	
4.	Is there a government food allowance and / or a financial incentive for patients with MC?	1.0	
5.	Is there a GF certification program for manufactured products for people with GD?		0
6.	Does the country have a national MC association?		0
	Total	1.5	

The total public policy score for celiac disease (PPSCD) obtained/ calculating for the Republic of Moldova is 1.5 points, which is categorized as low.

## Discussion

Governments attach great importance to ensuring food security and developing a wide variety of mechanisms for it. Despite budgetary constraints, the Republic of Moldova has numerous institutions involved in food security. However, the organizational structure does not fully reflect modern approaches to the delimitation of tasks between the authorities involved. And this often leads to deficiencies, especially in terms of food and nutrition security of people, for whom nutrition therapy is imperative.

### Does the Republic of Moldova have regulations regarding packaged industrial food products for people with CD?

The role of the regulatory authority in food labeling is to define the categories of information that should always be stated on a label and to provide an appropriate framework for the control of voluntary labeling (33). About 40.6% of all who member countries have regulations regarding gf industrial food products, this being the criterion, with the highest score, accumulated in the evaluation of the level of assistance of people with CD (22). The Republic of Moldova became a member of the world health organization in 1992, the cooperation within the who being carried out based on the biennial cooperation agreements. In 2018, the Republic of Moldova was elected as a member of the standing committee of the regional office of the world health organization. The choice is a recognition of the progress made by country in implementing the health policies recommended by the who, for the purpose of universal access to public health services (34). However, the Republic of Moldova does not have public policies on packaged industrial food gf products. The government decision, referring to the approval and implementation of the sanitary norms on nutrition labeling and labeling of special purpose foods, specifies only the need to indicate on the label the presence or absence of gluten, if the product is for children under 6 months of age. In the respective government decision, the inclusion on the label of foods and ingredients known to cause hypersensitivity is mandatory, regardless of their quantity in the product (35).

### Does the country have regulations regarding unpackaged meals and food for people with CD?

In the law on consumer information on food, the keyword–gluten, can be found only in the context of requesting the indication of information (voluntarily) on the absence or reduced presence of gluten in food and, stating (in the annex) that it can be found in certain grains and may cause allergies or intolerances (36). No labeling restrictions are specified for gf products, such as in Canada or Argentina (37). Australia and New Zealand have the toughest labeling laws in the world and apply to all food sold or prepared for sale, including imported food (38, 39). The New Zealand food standards code requires that: foods labeled “gluten-free” must not contain detectable gluten; and without oats or their products; or gluten-containing cereals that have used malt or their products. Foods labeled as “low in gluten” must contains no more than 20 mg gluten/100 g of the food (40).

Following a GFD can be difficult and can lead to social constraints due to the fear of gluten exposure outside one's own household. It is known that adherence to diet differs from one patient to another, with non-compliance ranging from 25 to 50% in children and adolescents. Factors leading to low compliance may be financial, cultural or psychosocial (15). Many countries support their citizens by developing public policies regarding meals and unpackaged foods for people with CD. In the Republic of Moldova there are no regulations regarding unpackaged meals and foods for coeliacs. Local pre-school institutions are not prepared to provide assistance to children with permanent gluten intolerance and provide them with food options. The problem of a GF alternative also arises in the case of school canteens and hospitals. For parents with children with CD, the lack of information in schools and kindergartens is a serious challenge. Local catering establishments (restaurants, catering services) do not provide GF menus to consumers. Even when, for commercial purposes, it is stated that the products are GF, they are not certified and are not controlled. It is only based on the presumption that the food was prepared from GF raw materials. This is largely due to the low level of awareness and knowledge of food service staff, lack of guidelines, etc. In Italy, for example, the Italian Celiac Association and the government have done an excellent job of educating restaurants on how to treat CD (41). There are even gluten-free meals in schools, hospitals and public food establishments (42).

### Is there a specialized healthcare service for celiac patients?

The incidence of CD in the Republic of Moldova varies from region to region, with an increasing trend due to the increased number of newly diagnosed cases. According to data taken from the national health management center, in 2019, for every 10,000 inhabitants, 63.8 people were diagnosed with CD. This is supposed to be the only visible part of the glacier. In support of the specialized health care service for celiac patients, the working group of the ministry of health of the Republic of Moldova (MoH RM) developed the national protocol for children with celiac disease, which represents the matrix for the development of institutional protocols. The protocol includes levels of primary care, outpatient and inpatient medical care, diagnostic algorithms, and a brief guide to the celiac patient (43). Currently, in the Republic of Moldova, the study of the problem of CD is being studied in children within the CD skills Interreg Danube transnational program funded by the EU, which is being implemented by Nicolae Testemi?anu State University of Medicine and Pharmacy, in the period 2020–2022. At present, no national data are known that could reflect the real situation of the total number of celiac patients (Adults, Children). There is no separate statistical record (15). The current national protocol on CD refers only to children and will be updated by the CD SKILS Project implementation team by the end of 2022 based on recommendations updated by the European Society of Gastroenterology, Hepatology and Nutrition. Studies about celiac disease in adults in the Republic of Moldova are not available. The nominated project highlighted only the number of children with CD who are registered with pediatric gastroenterologists but does not include research on the number of adult patients. Specialized medical care for patients with CD in the Republic of Moldova is provided based on the international recommendations provided for pediatric gastroenterologists, at the outpatient and hospital level, based on adjusted international and national protocols, in accordance with applicable law. A separate medical service for the care of patients suffering from CD is not in the Republic of Moldova.

### Is there a government food allowance and/or a financial incentive for patients with CD?

Each national health system has a different structure and approach to patients' rights, which includes different types of care in the information system, financial incentives, and additional medical services. The institutional clinical protocol of 2012, referring to CD in children in the Republic of Moldova states, according to the order of the Ministry of Health of the Republic of Moldova, that children with celiac disease are established the second degree of disability (44). The determination of the degree of disability in children with CD is performed based on the data of the complex examination (clinical, immune-serological, morpho-histological, genetic) (45). Patients diagnosed with CD receive financial allowances approved by the Government, according to the general recommendations for disability and can benefit, once a year from rehabilitation (46). In Romania, Bulgaria, Austria, etc., patients with CD do not benefit from free rehabilitation services (15). In that context, the answer to this question, being an affirmative one, was allocated 1 point.

### Is there a GF certification program for manufactured products for people with GD?

In the Republic of Moldova, the design, creation, and production of GF flour foods comes more from research and very little from industry, because investments in manufacturing lines, separate from milling and wheat baking, are too high (13). Another major challenge is the lack of a national certification program, which makes it difficult for manufacturers to design GF products and maintain their safety. At the same time, the lack of a national association for CD makes it more difficult to adopt a certification program for local GF Crossed Grain products, which is developed and operated by the Association of European Celiac Society (AOECS), an independent, non-profit organization.

### Does the country have a national CD association?

The role of CD associations is to respond to the need for clarity, honesty, and competence for everything related to this pathology. They can help improve the quality of life for people with CD and their families. CD associations forms and maintains dialogues with institutional interlocutors, bringing patients' voices to roundtables and debates on issues that concern them closely, dealing with media relations, responding to the need for dialogue with others, providing solutions to their problems etc. (32). In the Republic of Moldova there is no national association for coeliacs and no organized environment for this category of patients, there are no places to eat GF menus. In Romania, the Romanian Association for Gluten Intolerance (RAGI)—the only national association in Romania, accredited by the Association of European Celiac Society (AECS) and supported by the celiac disease management center, is helping people with gluten-related disorders. Only 34.4% of WHO member states have celiac associations, and this highlights the relevance of this criterion in improving the level of care for people with gluten-related disorders (22).

On average, the average level of care for people with CD on the European continent is the highest–3.63, followed by South America-2.86 and North America-1.05. Only six European countries reached the maximum score (France, Italy, the Netherlands, Slovenia, Sweden and the United Kingdom) (22). The PPSCD obtained for the Republic of Moldova is 1.5 points, which is categorized as low. The obtained results reflect the food security of people with disorders related to gluten consumption in the Republic of Moldova.

## Conclusions

- The review of national policies and investments to ensure the food security of people with gluten-related disorders practically elucidated and highlighted the major gaps and challenges for all the researched question tool.
- The development of policies, which consider both children and adults with CD, is current and necessary at all levels: legislative, educational, training and community support, etc.
- The academic environment, the food and medical sector, governance, could contribute to the development of PP and, respectively, to ensuring the food security of people with disorders related to gluten consumption by disseminating and popularizing research results, by educating and sensitizing the population, by integrating nutrition objectives for this population category, through social events, workshops, advice for the business environment, etc.
- Applying a holistic and personalized approach that would include, on the one hand, the celiac and his family, the doctor, the dietician, and the celiac support group, and on the other hand, understanding quality of life issues, using current evidence-based information and resources, monitoring GF product compliance and nutritional status of people with CD, could help improve quality of life, as well as economic impact at the level of the country.

## Data availability statement

The original contributions presented in the study are included in the article/Supplementary material, further inquiries can be directed to the corresponding author/s.

## Author contributions

All authors listed have made a substantial, direct, and intellectual contribution to the work and approved it for publication.

## Funding

This research was funded by the postdoctoral grant: Contributions regarding nutritional eradication of gluten consumption diseases, nr. 21.00208.5107.06/PD, and the state project Personalized nutrition and intelligent technologies for my well-being, nr. 20.80009.5107.10/PS. Both funded by the National Agency for Research and Development (NARD).

## Conflict of interest

The authors declare that the research was conducted in the absence of any commercial or financial relationships that could be construed as a potential conflict of interest.

## Publisher's note

All claims expressed in this article are solely those of the authors and do not necessarily represent those of their affiliated organizations, or those of the publisher, the editors and the reviewers. Any product that may be evaluated in this article, or claim that may be made by its manufacturer, is not guaranteed or endorsed by the publisher.
