# Supplementary material for: Food security of people with celiac disease in the Republic of Moldova through prism of public policies
Source: Front Public Health. 2022 Oct 3;10:961827. doi: 10.3389/fpubh.2022.961827 (PMC9574389; doi:10.3389/fpubh.2022.961827)
Supplement: Supplementary file 1 [file Data_Sheet_1.docx]

**Figure 1. Dimensions of food security** (1), (2)

**Figure 2. Disorders related to gluten consumption** (8)

**Figure 3. International standards governing gluten-free products**
